# Dysregulated Iron Metabolism-Associated Dietary Pattern Predicts an Altered Body Composition and Metabolic Syndrome

**DOI:** 10.3390/nu11112733

**Published:** 2019-11-11

**Authors:** Anggun Rindang Cempaka, Sung-Hui Tseng, Kuo-Ching Yuan, Chyi-Huey Bai, Alexey A. Tinkov, Anatoly V. Skalny, Jung-Su Chang

**Affiliations:** 1 Department of Nutrition Science, Faculty of Medicine, Brawijaya University, Malang 65145, Indonesia; anggunrindangcempaka@gmail.com; 2 School of Nutrition and Health Sciences, College of Nutrition, Taipei Medical University, Taipei 110, Taiwan; 3 Department of Physical Medicine and Rehabilitation, Taipei Medical University Hospital, Taipei 110, Taiwan; m003089010@tmu.edu.tw; 4 Department of Physical Medicine and Rehabilitation, School of Medicine, College of Medicine, Taipei Medical University, Taipei 110, Taiwan; 5 Department of Emergency and Critical Care Medicine, Taipei Medical University Hospital, Taipei 110, Taiwan; traumayuan@gmail.com; 6 Department of Public Health, College of Medicine, Taipei Medical University, Taipei 110, Taiwan; baich@tmu.edu.tw; 7 Department of Public Health, College of Public Health, Taipei Medical University, Taipei 110, Taiwan; 8 Department of Medical Elementology, Peoples’ Friendship University of Russia (RUDN University), Moscow 117198, Russia; tinkov.a.a@gmail.com (A.A.T.); skalnylab@gmail.com (A.V.S.); 9 Laboratory of Biotechnology and Applied Bioelementology, Yaroslavl State University, Yaroslavl 150003, Russia; 10 Laboratory of Molecular Dietology, IM Sechenov First Moscow State Medical University, Moscow 119146, Russia; 11 Graduate Institute of Metabolism and Obesity Sciences, College of Nutrition, Taipei Medical University, Taipei 110, Taiwan; 12 Nutrition Research Center, Taipei Medical University Hospital, Taipei 110, Taiwan; 13 Chinese Taipei Society for the Study of Obesity (CTSSO), Taipei 11031, Taiwan

**Keywords:** central obesity, dysregulated iron metabolism, hepcidin, ferritin, dietary pattern, visceral fat, skeletal muscle mass, metabolic syndrome

## Abstract

Diet plays an important role in the development of obesity and may contribute to dysregulated iron metabolism (DIM). A cross-sectional survey of 208 adults was conducted in Taipei Medical University Hospital (Taipei, Taiwan). A reduced-rank regression from 31 food groups was used for a dietary pattern analysis. DIM was defined as at least four of the following criteria: serum hepcidin (men >200 ng/mL and women >140 ng/mL), hyperferritinemia (serum ferritin of >300 ng/mL in men and >200 ng/mL in women), central obesity, non-alcoholic fatty liver disease, and two or more abnormal metabolic profiles. Compared to non-DIM patients, DIM patients were associated with an altered body composition and had a 4.52-fold (95% confidence interval (CI): (1.95–10.49); *p* < 0.001) greater risk of metabolic syndrome (MetS) after adjusting for covariates. A DIM-associated dietary pattern (high intake of deep-fried food, processed meats, chicken, pork, eating out, coffee, and animal fat/skin but low intake of steamed/boiled/raw foods and dairy products) independently predicted central obesity (odds ratio (OR): 1.57; 95% CI: 1.05–2.34; *p* < 0.05) and MetS (OR: 1.89; 95% CI: 1.07–3.35; *p* < 0.05). Individuals with the highest DIM pattern scores (tertile 3) had a higher visceral fat mass (%) (β = 0.232; 95% CI: 0.011–0.453; *p* < 0.05) but lower skeletal muscle mass (%) (β = −1.208; 95% CI: −2.177–−0.239; *p* < 0.05) compared to those with the lowest DIM pattern scores (tertile 1). In conclusion, a high score for the identified DIM-associated dietary pattern was associated with an unhealthier body composition and a higher risk of MetS.

## 1. Introduction

Obesity is a major public health problem which has substantially increased worldwide in recent decades. In Taiwan, the prevalence of being overweight and obese among adult is 43.4% according to the 2013–2014 Nutrition and Health Survey [1]. A recent meta-analysis showed that iron homeostasis is affected by obesity [2]. Dysregulated iron metabolism (DIM) was first observed by Moirand and Deugnier in 1997, who described a new clinical symptom of hepatic iron overload in patients without hemochromatosis (HFE) [3]. Recently, DIM was indicated in a wide range of diseases including cancer [4], polycythemia vera [5], and skeletal muscle and nerve degeneration [6]. Dysmetabolic iron overload syndrome (DIOS), a more-precise clinical definition for hepatic iron accumulation, is a condition characterized by mild liver iron overload, elevated serum ferritin and hepcidin, but normal or low serum iron/transferrin saturation (TS) together with the presence of metabolic disorders such as obesity, insulin resistance (IR), hypertension, and dyslipidemia [7,8]. Deugnier and colleagues estimated that 34.5–51.5% of patients with non-alcoholic fatty liver disease (NAFLD) had DIOS [8].

Obese people are reported to have excessive visceral fat accumulation (also known as abdominal or central obesity) and have a higher risk of metabolic syndrome (MetS) [5]. Several population-based surveys showed a strong link between elevated serum ferritin levels and a risk of MetS [3,9], and this observation was confirmed in a systemic review and meta-analysis [10]. Elevated serum ferritin (also known as hyperferritinemia) not only reflects hepatic iron levels [11] but also indicates an increased level of adipose tissue inflammation [12] or visceral fat mass [13], and impaired skeletal muscle mass [6]. Hence, DIM may directly contribute to the pathology of obesity-related metabolic complications through alterations in the body composition.

Elevated serum hepcidin concentrations are considered to be the underlying cause of DIM [7,8]. Hepcidin is a peptide hormone mainly released by hepatocytes and which acts as a negative iron regulator [14]. Generally, hepcidin maintains systemic iron homeostasis by controlling the duodenal absorption of iron and the release of stored iron into plasma [15]. Hepcidin is stimulated by multiple factors, including the body’s iron status (plasma iron, hypoxia, and anemia), erythropoiesis activity, hormones (e.g., estrogen and leptin), and inflammation [7,14,16]. The main role of hepcidin is to regulate the only known mammalian iron exporter, ferroportin [14]. Elevated hepcidin expression leads to ferroportin degradation, resulting in elevation of iron concentrations within cells and low circulating iron levels [17]. On the other hand, a lack of hepcidin expression results in elevation of ferroportin expression which enhances the iron absorption rate [17].

Dietary factors play important roles in the development of obesity and may contribute to an iron imbalance. Studies showed that meat, fish, and poultry are positively associated with the serum iron status, while dairy products, bread, cereals/fiber, protein-based foods/soy protein, tea, and coffee are negatively correlated with iron status [18]. However, meat, particular red meat, is part of the Western diet (characterized by high intake of red meat, processed meats, refined carbohydrates, and fatty foods but low intake of vegetables, fruits, and whole grains), and Pacheho et al. reported that red meat consumption was positively correlated with ferritin levels and the risk of myocardial infarction (hazard ratio (HR) per 50 g of daily intake: 1.18; 95% confidence interval (CI): (1.05–1.33) [19]. A study by Sabrina et al. reported that young adult women who consumed an iron: ferritin ratio dietary pattern (high intake of beef, lamb, dairy products, fruits, and whole grains but low intake of refined carbohydrates and deep- and stir-fried foods) were associated with a healthy body composition and a 90% reduced risk of fatty liver progression (odds ratio (OR): 0.10; 95% confidence interval (CI): 0.02–0.47; *p* < 0.001) [13].

Currently, the role of diet in the development of DIM remains unclear. A reduced-rank regression (RRR) analysis, a data-driven (exploratory) method to characterize major patterns of food intake, is regarded as an approach to dissect the link between diet and chronic diseases [20]. The broad aim of this study was to investigate dietary patterns that are associated with DIM and their predictive effects on the body composition and MetS in 208 Taiwanese adults. Specifically, we aimed to: (1) identify dietary pattern scores associated with concentrations of serum biomarkers related to DIM using an RRR analysis and (2) to test whether the dietary pattern obtained was prospectively associated with the body composition, central obesity, and MetS.

## 2. Materials and Methods

### 2.1. Participants

This study was conducted in accordance with the Declaration of Helsinki (1964) and its later amendments (2013). The study protocol was reviewed and approved by the Taipei Medical University Institutional Ethical Review Committee (TMU-JIRB 201502018). Written informed consent was signed by all eligible subjects. Briefly, this was a cross-sectional study, and the sampling method was a non-probability sampling. Taiwanese adults (*n* = 230) aged 20–65 years were enrolled through the recruitment process at the Division of Gastroenterology and Hepatobiliary Disease, Department of Internal Medicine, Taipei Medical University Hospital (Taipei, Taiwan), from July 2015 to June 2016. The sample size was calculated based on studies by Deugnier et al. [8] and Dongiovanni et al. [21]. With a two-sided significance level of 95% and a power of 80%, the calculated sample size was 32 per group. Twenty-two subjects were excluded from the study because 12 persons had one of the following criteria: (1) a hepatitis virus (HV) infection history (e.g., HVA, HVB, or HVC) or liver malignancy; (2) thalassemia, polycystic ovary syndrome, or drug-induced hepatitis history; (3) excessive alcohol intake (alcohol intake >20 g/week for women or >30 g/week for men); (4) pregnancy or breastfeeding; and (5) use of hormone-replacement therapy or iron supplementation. One person declined to participate, and nine persons did not have blood data. Only 208 subjects (105 men and 103 women) were eligible and with no missing data were included in the final analysis. The reporting of this study conformed to the Strengthening the Reporting of Observational Studies in Epidemiology (STROBE) and Enhancing the Quality and Transparency of Health Research (EQUATOR) guidelines (Supplementary Table S1) [22,23].

### 2.2. Definitions

Currently, there is still no consensus for DIM diagnostic criteria. However, there are some clinical features that are associated with DIM, such as alterations in iron and metabolic profiles. Therefore, we defined DIM as the presence of at least four of the following criteria: (1) elevated serum hepcidin (>200 ng/mL in men and >140 ng/mL in women); (2) hyperferritinemia (serum ferritin (SF) >300 ng/mL in men and >200 ng/mL in women) [14]; (3) central obesity (waist circumference (WC) of ≥90 cm in men and ≥80 cm in women) [24,25]; (4) NAFLD; and (5) the presence of at least two abnormal metabolic profiles (1) a fasting triglyceride (TG) level of ≥150 mg/dL; (2) a high-density lipoprotein cholesterol (HDL-C) level of <40 mg/dL in men and <50 mg/dL in women; (3) systolic blood pressure (BP) of ≥130 mmHg or diastolic BP of ≥85 mmHg; and (4) a fasting plasma glucose (FPG) concentration of ≥100 mg/dL. Abdominal ultrasound was used to screen individuals with NAFLD based on the following criteria: parenchymal brightness, liver-to-kidney contrast, deep-beam attenuation, bright vessel walls, and gallbladder wall definition. An abnormal serum alanine aminotransferase (ALT) level was defined as >40 U/L. Anemia was defined as hemoglobin (Hb) of <13 g/dL in men and <12 g/dL in women. Iron-deficiency anemia (IDA) was defined as two or more indicators of the iron status being abnormal: (1) Hb of <13 g/dL in men and <12 g/dL in women; (2) SF of <12 µg/L; and (3) TS of <15% [14,26]. MetS was determined based on the modified National Cholesterol Education Program Adult Treatment Panel III for the Asia Pacific [27,28]: with three of more of the following criteria: fasting TG of ≥150 mg/dL; HDL-C of <40 mg/dL in men and <50 mg/dL in women; systolic BP of ≥130 mmHg or diastolic BP of ≥85 mmHg; and an FPG of ≥100 mg/dL [14]. Dyslipidemia was defined when a subject had at least one of the following criteria: (1) TG level of ≥200 mg/dL; (2) total cholesterol (TC) level of ≥240 mg/dL; (3) HDL-C level of <35 mg/dL; (4) low-density lipoprotein (LDL)-C level of ≥160 mg/dL; (5) TC/HDL-C ratio of ≥5; and (6) use of lipid-lowering medicine [14]. Diabetes mellitus (DM) was defined when a subject had at least one of the following criteria: (1) an FPG concentration or ≥126 mg/dL and (2) glycated Hb (HbA1C) of ≥6.5% [14].

### 2.3. Questionnaires

A basic questionnaire was used to obtain general information about the subjects, including age, sex, nationality, anthropometric data, health status/medical history, medication use, and alcohol consumption. A modified self-reporting food frequency questionnaire (FFQ) based on the Chinese version of the FFQ for the Taiwanese population [29] was used to evaluate: (1) the usual weekly intake frequency of 66 food items which were grouped into 31 food groups according to similarities; (2) five types of commonly used cooking methods; and (3) the frequency of eating out. The self-report FFQ consisted with eight scales: (1) ≤1 times/week; (2) 2 or 3 times/week; (3) 4 or 5 times/week; (4) 6 or 7 times/week; (5) 8~10 times/week; (6) 11~13 times/week; (7) 14~16 times/week; and (8) ≥17 times/week. To reduce the information bias, the content validity of the modified FFQ was assessed by three nutrition experts. The internal consistency/reliability of the modified FFQ was high with Cronbach’s alpha coefficient of 0.85.

### 2.4. Anthropometric Measurements

Body weight (BW), height, and WC were measured for each subject by the investigator. The body-mass index (BMI) was determined as the BW divided by body height squared (kg/m^2^). WC was measured at the midpoint between the bottom rib and the top of the hip bone (iliac crest). A direct segmental multi-frequency bioelectrical impedance analysis (DSM-BIA) was conducted using a DSM-BIA meter X-SCAN Plus-II analyzer (Jawon, Seoul, Korea).

### 2.5. Laboratory Measurements

Fasting blood was collected from overnight-fasted participants. Briefly, blood tests included a complete blood cell count, glucose and lipid profiles, liver injury and oxidative stress biomarkers, and serum iron biomarkers. Fasting plasma insulin was assessed by a radioimmunoassay (Millipore, Billerica, MA, USA). FPG was quantitated by the glucose oxidase method. Serum TC, TG, and HDL-C were determined using reagents from Beckman Laboratories and quantitated by an autoanalyzer (Beckman Coulter Unicel DxC 800, Taipei, Taiwan). The LDL-C level was calculated using the Friedewald formula (LDL-C = TC – (HDL-C + TG/5) mg/dL). Hb was measured by the direct cyanometahemoglobin method, which is the gold standard (Merckotest, Merck, Taipei, Taiwan). A ferrozine-based colorimetric method was used to quantitate serum iron. SF was assessed by an electrochemiluminescence immunoassay and was quantitated with a Roche Modular P800 analyzer (Roche; Taipei, Taiwan). Serum hepcidin was assessed by an enzyme-linked immunosorbent assay (ELISA) according to the manufacturer’s instructions (DRG International; Springfield Township, NJ, USA). Percentage TS (%TS) was calculated as (serum iron/total iron-binding capacity (TIBC)) × 100%. HbA1C was assessed by an affinity high-performance liquid chromatography (HPLC) method. Serum ALT was measured by a colorimetric method with the Beckman DxC 800 (Beckman Coulter). The serum *malondialdehyde* (MDA) concentration, a lipid peroxidation marker, was determined based on the reaction of MDA with thiobarbituric acid to form *thiobarbituric acid*-reactive species. Serum nitric oxide (NO) was assessed with the Griess reagent (Sigma-Aldrich, Taipei, Taiwan).

### 2.6. Statistical Analysis

**Figure 1:**
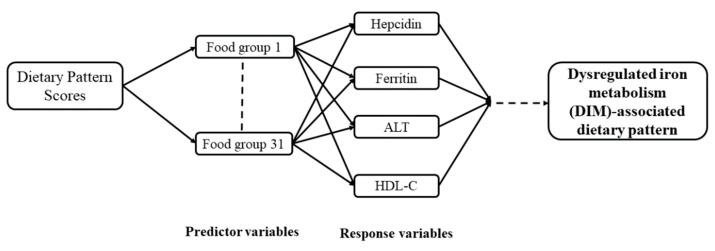
Direct acyclic graph of the reduced-rank regression (RRR) conceptual framework. ALT, alanine aminotransferase; HDL-C, high-density lipoprotein cholesterol.

Statistical analyses were performed using the IBM SPSS statistics software vers. 20 (IBM, Armonk, NY, USA) and SAS 9.4 (SAS Institute, Cary, NC, USA). Categorical data are presented as the number (percentage (%)), and continuous data are presented as the mean ± standard deviation (SD). A general linear model was used to analyze the *p* for trend. Multiple comparison analysis testing in a one-way analysis of variance (ANOVA) (with the Bonferroni post-hoc correction) was used to compare multiple groups. A chi-squared test was used to compare categorical variables. An RRR analysis was applied to derive DIM-associated dietary patterns. The RRR was originally described by Hoffmann and colleagues to delineate the relationship between diet and chronic diseases [20]. This study used 31 food groups from the FFQ as predictor variables. Serum hepcidin, ferritin, ALT, and HDL-C were selected as response variables based on their strong correlations with DIM. For dietary pattern scores, only food groups that had factor loadings greater than or equal to positive/negative 0.20 (≥|0.20|) were selected. Factor loading indicates the correlation between a food group and the RRR-derived dietary pattern. A dietary pattern score was calculated for each participant by deriving a score that represents the sum of food intake variables weighted by factor loadings to reflect their intake conformity with the DIM-associated dietary pattern. A multivariate logistic regression was performed to identify risk factors associated with DIM and determine the role of the dietary pattern score in predicting the risk of DIM and MetS. A multivariate linear regression was used to evaluate the relationship between DIM-associated dietary pattern scores and body composition. The direct acyclic graph in Figure 1 explains the conceptual framework of the RRR. A *p* value of ≤0.05 was considered statistically significant for all analyses. The figure was created using GraphPad Prism vers. 5 (GraphPad Software, La Jolla, CA, USA).

## 3. Results

### 3.1. Baseline Characteristics of the Study Population According to Dysregulated Iron Metabolism (DIM)

Table 1 shows baseline characteristics of study participants according to their DIM status. Mean ages were 40.55 ± 1.01 years for non-DIM subjects and 47.15 ± 1.58 years for DIM patients (*p* = 0.001). Prevalence rates of DIM and MetS were 22.6% and 24.5%, respectively. In general, DIM patients were older and heavier, and had a higher incidence of metabolic diseases (e.g., NAFLD, MetS, central obesity, DM, dyslipidemia, and hypertension), higher levels of circulating iron (e.g., Hb, serum hepcidin, and SF), liver injury and oxidative stress biomarkers (NO, MDA, and ALT), blood lipids (high TG and LDL-C but low HDL-C), and glucose biomarkers (FPG and HbA1C) compared to non-DIM subjects (all *p* < 0.05; Table 1). In addition, anthropometric measurements showed that DIM patients were associated with a higher BMI, WC, body fat mass, and visceral fat mass but lower skeletal muscle mass compared to non-DIM patients (all *p* < 0.001; Table 1).

### 3.2. DIM and Risk of Altered Body Composition and Metabolic Syndrome (MetS)

**Figure 2:**
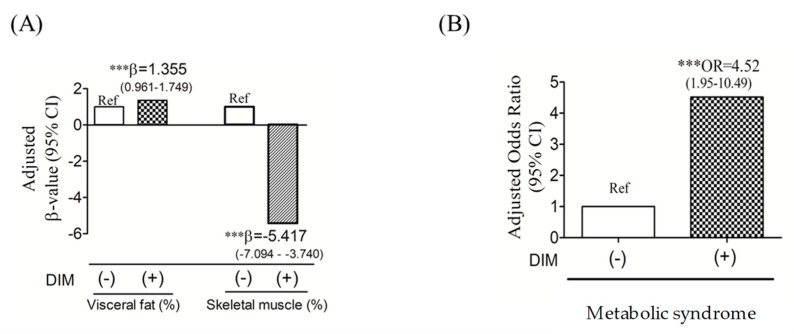
Adjusted multivariate linear and logistic regression and 95% confidence intervals of dysregulated iron metabolism (DIM) in terms of visceral fat mass (%) and skeletal muscle mass (%) (**A**) and metabolic syndrome (**B**). (**A**) the Beta coefficient (β-value) was adjusted by age and sex, and (**B**) odds ratio (OR) was adjusted by age, sex, and body-mass index (BMI) (*N* = 208). *** *p* ≤ 0.001.

We next performed a multivariate linear and logistic regression analysis to evaluate relationships among DIM, body composition, and MetS. Figure 2A shows that compared to non-DIM individuals, individuals with DIM were associated with increased % visceral fat mass (β = 1.355 (0.961–1.749), *p* < 0.001) and decreased % skeletal muscle mass (β = −5.417 (−7.094–−3.740), *p* < 0.001) after adjusting for age and sex. A multivariate logistic regression analysis showed that compared to non-DIM subjects, DIM patients had 4.52 (OR: 4.52; 95% CI: 1.95–10.49; *p* < 0.001) higher risks of developing MetS after adjusting for age, gender, and BMI (Figure 2B).

### 3.3. DIM-Associated Dietary Pattern Scores by the Reduced-Rank Regression (RRR)

Currently, dietary risk factors associated with DIM remain undefined. We applied an RRR to derive a predictive dietary pattern for DIM. Response variables for the RRR were selected based on relationships between independent variables and DIM. A multivariate logistic regression analysis revealed that serum hepcidin (OR = 1.018 (1.010–1.025); *p* ≤ 0.001), SF (OR = 1.007 (1.002–1.012); *p* = 0.005), and ALT (OR = 1.029 (1.004–1.055); *p* = 0.021) were positively correlated with DIM, while HDL-C (OR = 0.929 (0.865–0.999); *p* = 0.046) was negatively correlated with DIM (Table 2).

We next investigated relationships of selected response variables (hepcidin, ferritin, ALT, and HDL-C) with the visceral fat mass and skeletal muscle mass. The multivariate linear regression analysis found positive trends between the percent visceral fat mass and hepcidin (β = 0.001; 95% CI: 0.0001–0.002; *p* < 0.05), and ALT (β = 0.004; 95% CI: 0.001–0.007; *p* < 0.05) after adjusting for age, sex, and the BMI (Table 3, model 3). In contrast, the skeletal muscle mass was positively correlated with HDL-C (β = 0.032; 95% CI: 0.004–0.060; *p* < 0.05) and negatively correlated with hepcidin (β = −0.004; 95% CI: −0.008–−0.0001; *p* < 0.05 and ALT (β = −0.020; 95% CI: −0.035–−0.004; *p* < 0.05) after adjusting for age, sex, and the BMI (Table 3, model 3).

Table 4 shows percentages of food variations explained by the first dietary pattern scores and factor loadings of the food groups. The first dietary pattern derived by the RRR was characterized by high frequencies of deep-fried foods, processed meats, chicken, pork, eating out, coffee, and animal fat/skin but low intake frequencies of steamed/boiled/raw foods and dairy products (factor loadings ≥ |0.20|) (Table 4). This dietary pattern explained 64.73% of the total variation in the food groups.

### 3.4. Relationships among DIM-Associated Dietary Pattern Scores, MetS, Central Obesity, and Body Composition

Table 5 shows clinical characteristics and blood biochemical levels according to tertiles of dietary pattern scores. Participants with higher dietary pattern scores were more likely to be male, have central obesity (e.g., higher WC and visceral fat mass), have higher incidences of metabolic diseases (e.g., MetS or high TG but low HDL-C), and have higher levels of circulating iron (e.g., Hb, serum hepcidin, and ferritin) and the liver injury biomarker (ALT) (Table 5).

**Figure 3:**
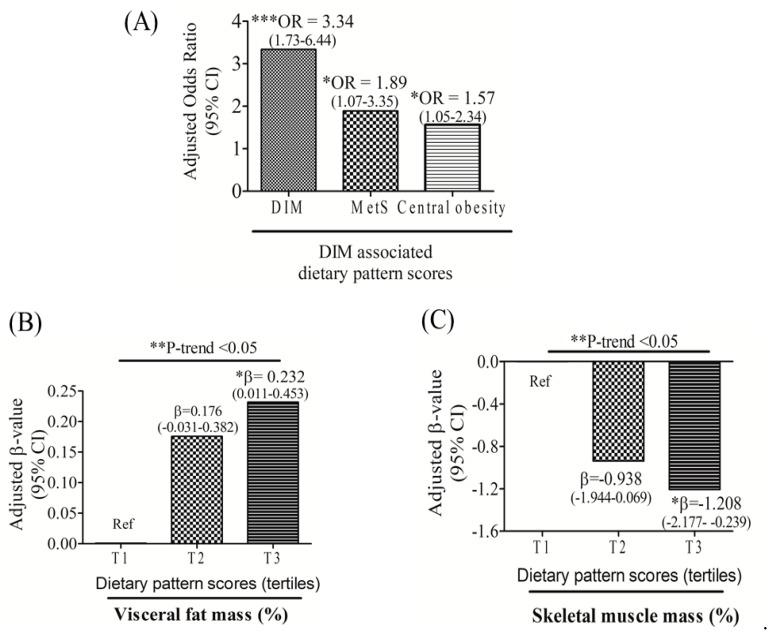
Multivariate logistic analysis adjusted for age, gender, and body-mass index and linear regression and 95% confidence intervals (CIs) of dysregulated iron metabolism (DIM)-associated dietary pattern scores for predicting DIM (**A**), metabolic syndrome (MetS) (**A**), central obesity (**A**), visceral fat mass (%) (**B**), and skeletal muscle mass (%) (**C**). * *p* < 0.05; ** *p* < 0.01; *** *p* ≤ 0.001.

The multivariate logistic regression analysis showed that DIM-associated dietary pattern scores independently predicted DIM (OR: 3.34; 95% CI: 1.73–6.44; *p* < 0.001), MetS (OR: 1.89; 95% CI: 1.07–3.35; *p* < 0.05), and central obesity (OR: 1.57; 95% CI: 1.05–2.34; *p* < 0.05) after adjusting for age, sex, and BMI (Figure 3A). We next investigated relationships between DIM-associated dietary pattern scores and body composition. A multivariate linear regression analysis adjusted for age, sex, and BMI showed that individuals with the highest DIM pattern scores (tertile 3) had increased visceral fat mass (%) (β = 0.232; 95% CI: 0.011–0.453; *p* < 0.05) but decreased skeletal muscle mass (%) (β = −1.208; 95% CI: −2.177–−0.239; *p* < 0.05) compared to those with the lowest scores (tertile 1) (Figure 3).

## 4. Discussion

To our knowledge, this is the first study to investigate dietary risk factors associated with DIM. Our results suggest that individuals who tend to eat out and that have a preference for a Western diet (indicated by high intake frequencies of meat, coffee, and fatty and deep-fried foods and low intake of steamed/boiled/raw foods) are more likely to develop an unhealthy body composition and DIM, which in turn, may promote progression of obesity to MetS. A nutritional iron deficiency occurs when an insufficient amount of dietary iron is absorbed to meet the requirements of the body. Although a Western diet is rich in meat, which contains bioavailable heme iron, it is also associated with increased fat intake. Chang et al. showed that overweight and obese women who consumed a “high-fat, low-carbohydrate” diet had a 10-fold higher risk of developing iron-deficiency anemia (IDA; odds ratio (OR): 10.12; 95% CI: 1.27–80.79; *p* = 0.017) [30]. Increased intake of dietary fat but a low intake of steamed/boiled/raw foods (e.g., vegetables) may increase systemic inflammation and promote the pathophysiology of DIM [7,12,17].

The DIM-associated dietary pattern explained 64.7% of the total variation in food groups and 36.5% of the total variation in response variables. In this study, we used four response variables (SF, hepcidin, ALT, and HDL-C) because of their strong correlations with DIM. Among these response variables, the DIM-associated dietary pattern was largely explained by SF (which explained 14.6% of the variation in dietary pattern scores), hepcidin (which explained 8.9% of the variation), ALT (which explained 8.8% of the variation), and to a lesser extent, serum HDL (which explained 4.3% of the variation). Numerous studies have shown that elevated SF concentrations and body iron stores are strong risk factors for visceral fat mass and metabolic disorders [3,26,27,28]. Moreover, excess adiposity can increase hepcidin secretion, which possibly acts through proinflammatory cytokines or leptin or IR, and elevated serum hepcidin may result in a lower iron absorption rate and hypoferremia [7,8,17,29].

Our study found that SF was positively associated with the consumption of processed meats (β = 18.65; 95% CI: 0.03–37.27; *p* = 0.05) and negatively associated with steamed/boiled/raw food intake (β = −13.87; 95% CI: −27.75–0.02; *p* = 0.05) (data not shown). Beck et al. found that high intake of red meat, poultry, fish, and vegetables was associated with higher levels of Hb, serum iron, and SF [18]. Avila et al. also reported a positive correlation between red meat intake and SF levels among Chilean men [31]. Our study also found that hepcidin was significantly associated with the consumption frequency of deep-fried foods (β = 24.31; 95% CI: 4.87–43.75; *p* = 0.015). Deep-fried foods can trigger hepatic hepcidin production via oxidative mediators (e.g., NO) and proinflammatory cytokines (e.g., tumor necrosis factor-α) [32]. HDL-C was inversely associated with the frequency of eating out (β = −2.74; 95% CI: −5.34–−0.14; *p* = 0.039). Cohen and Bhatia showed that eating out was associated with metabolic disorder diseases [33]. Foods purchased away from home tend to be energy-dense and prepared by deep-frying and not steaming/boiling compared to homemade foods [33,34].

Traditionally, a liver biopsy is the gold standard method for diagnosing hepatic iron overload. However, a liver biopsy is invasive and costly and occasionally may cause complications. The World Health Organization (WHO) suggests that, in the absence of HFE and inflammation, SF can serve as a surrogate marker for diagnosing iron overload (>200 μg/L in men or >150 * *μg/L in women) [35]. However, SF is sensitive to inflammation and might not truly reflect hepatic iron overload. Recently, non-invasive approaches, like magnetic resonance (MR) imaging, are being used for hepatic iron quantification, and it is regarded as a suitable substitute for SF levels and a liver biopsy. Branisso et al. examined 152 biopsy-proven NAFLD patients and found that 37% patients had DIOS. In addition, those authors showed that the cutoff point of the ferritin level for diagnosing hepatic iron overload using a liver biopsy was 284.3 ng/mL [36]. In the current study, we used gender-specific cutoff points for diagnosing hyperferritinemia (>300 ng/mL in males and >200 ng/mL in females). Based on this definition, the current study found only 55.3% of DIM patients with hyperferritinemia. We reasoned that the relatively low prevalence rate of hyperferritinemia may have been due in part to the difference in SF cutoff points. Future studies are needed to validate the cutoff point of SF for diagnosing hepatic iron overload in overweight and obese adults.

In the present study, the prevalence rate of DIM was 22.6% (22.9% in males and 22.3% in females). This rate is similar to that reported by Dongiovanni and colleagues [21] that 20%~30% of patients with NAFLD and MetS had DIM. In contrast, Deugnier et al. [8] reported that the prevalences of DIM in patients with NAFLD were 34.5% and 51.5%, respectively. This discrepancy may have been due in part to the lack of consensus on a clinical definition of DIM. Despite identifying certain clinical features, there is still no clinical cutoff point for a diagnosis of DIM. Deugnier and colleagues proposed diagnostic criteria for DIM based on three criteria: (1) normal or moderately increased TS (<60%); (2) the presence of metabolic disorders (high BMI, android distribution of fat, hypertension, dyslipidemia, and high glucose levels); and (3) excessive hepatic iron levels [8]. Dongiovanni et al. proposed that clinical features of DIM be defined by (1) the presence of obesity and metabolic alterations, (2) the presence of a fatty liver, (3) hyperferritinemia with normal or low serum iron, (4) mild increases in hepatic and body iron stores, and (5) the absence of ferroportin−1 mutations or polymorphisms [21]. In the present study, we defined DIM as the presence of at least four of the following criteria: (1) serum hepcidin (>200 ng/mL in men and >140 ng/mL in women), (2) hyperferritinemia (>300 ng/mL in men and >200 ng/mL in women), (3) central obesity, (4) NAFLD, and (5) the presence of at least two abnormal metabolic profiles. Currently, there is no definitive clinical cutoff point for abnormal values of serum hepcidin. In our study, mean concentrations of serum hepcidin for adult men and women were 184.91 ± 9.38 and 115.44 ± 10.34 ng/mL (*p* < 0.001), respectively. Mean serum hepcidin levels for normal-weight men and overweight/obese men were 165.39 ± 12.38 and 200.83 ± 13.47 ng/mL (*p* = 0.046) and 102.95 ± 13.06 and 140.33 ± 19.69 ng/mL (*p* = 0.200) for normal-weight and overweight/obese women, respectively. Based on these findings, we defined elevated serum hepcidin according to mean values of overweight/obese adult men and women of >200 and >140 ng/mL, respectively. Overall, despite differences in the diagnosis of DIM and the lack of information on liver iron levels, our study agrees with others in which DIM is more likely to be present in obese adults with metabolic disorders [7,8,21,26].

In this study, we used the RRR to derive DIM-associated dietary patterns. The RRR was chosen because RRR-derived dietary patterns are more likely to reflect dietary patterns associated with health outcomes compared to those derived from other dietary pattern analytical methods such as a principal component analysis (PCA) or factor analysis [20,37,38]. Using the RRR, we could determine which dietary patterns were associated with the development of diseases by combining prior information of disease biomarkers and dietary information from other studies [20,38]. Factors in the RRR were derived from both disease-specific biomarkers (responses) and food groups (predictors), while factors obtained from a PCA reflect only dietary habits of the population [39]. In the RRR, we chose serum hepcidin, ferritin, ALT, and HDL as response variables based on their strong correlations with DIM. So, dietary pattern scores were generated by taking into account both the power of predictors (food groups consumed in our study population) and response variables. In contrast, the PCA relies solely on inter-correlations among dietary variables, which might not fully reflect dietary qualities that are most relevant to specific disease etiologies [37]. A PCA or factor analysis was criticized for its subjectivity and for being poorly correlated with disease risks in some studies [20,40].

There are some limitations to this study. First, this was a cross-sectional study; therefore, causal relationships could not be evaluated. Second, there was a relatively small sample size (*n* = 208). Third, hyperferritinemia was used to indicate iron dysregulation and not hepatic iron levels by a liver biopsy. Liver biopsies are the gold standard for assessing tissue iron overload, but it would be difficult to perform them in a population-based study. Although SF is more suitable for population-based studies, obesity-related inflammation might also induce hyperferritinemia. Hence, SF is also regarded as an acute-phase reactant and is associated with a risk of MetS. Fourth, dietary patterns were assessed by the FFQ and not by 24-h dietary recall; therefore, we did not know the actual amount of food intake of participants. Fifth, the RRR was used to derive DIM-associated dietary pattern scores. The RRR method requires in-depth knowledge of diet–disease relationships in order to select appropriate response variables. Our study selected four response variables (hepcidin, ferritin, ALT, and HDL-C) based on their strong correlations with DIM. However, selecting response variables can be subjective, and this might have resulted in different dietary patterns in different age groups or studies.

## 5. Conclusions

Our results suggest that individuals who tend to eat out and that have a preference for a Western diet (indicated by high intake frequencies of meat, coffee, and fatty and deep-fried foods and low intake of steamed/boiled/raw foods) are more likely to develop an unhealthy body composition and DIM, which in turn, may promote the progression of obesity to MetS. Future research with a larger sample size or different populations is required to validate our current findings due to variations in the habitual consumption of different food groups.

## Figures and Tables

**Table 1:** Baseline characteristics of the study population according to the dysregulated iron metabolism (DIM) status (*N* = 208).

Characteristic	Study Population (*N* = 208)		*p* Value *
	DIM (−) (*n* = 161)	DIM (+) (*n* = 47)	
Age (years)	40.55 ± 12.76	47.15 ± 10.82	0.001
Gender (*n*, %)			
Male	81 (50.3)	24 (51.1)	0.928
Female	80 (49.7)	23 (48.9)	
NAFLD (*n*, %)	124 (77.0)	46 (97.9)	0.001
MetS (*n*, %)	22 (13.7)	29 (61.7)	<0.001
Central obesity (*n*, %)	54 (33.5)	45 (95.7)	<0.001
Diabetes mellitus (*n*, %)	15 (9.3)	10 (21.3)	0.027
Dyslipidemia (*n*, %)	32 (19.9)	28 (59.6)	<0.001
Hypertension (*n*, %)	69 (42.9)	35 (74.5)	<0.001
Abnormal ALT (*n*, %)	19 (11.8)	23 (48.9)	<0.001
Anthropometry			
BMI (kg/m^2^)	23.41 ± 4.37	28.59 ± 5.77	<0.001
Waist circumference (cm)	83.04 ± 11.80	96.86 ± 12.88	<0.001
Male	87.98 ± 9.32	101.63 ± 10.26	<0.001
Female	78.04 ± 11.98	91.88 ± 13.65	<0.001
Body fat mass (%)	26.47 ± 5.90	32.38 ± 5.53	<0.001
Skeletal muscle mass (%)	67.79 ± 5.83	61.93 ± 5.48	<0.001
Visceral fat mass (%)	3.37 ± 1.18	4.85 ± 1.23	<0.001
Glucose biomarkers			
Fasting blood glucose (mg/dL)	89.83 ± 16.35	97.36 ± 24.81	0.045
Insulin (mIU/mL)	9.62 ± 5.43	9.11 ± 6.08	0.327
HbA1C (%)	5.72 ± 0.83	6.16 ± 1.31	0.001
Lipid biomarkers			
Total cholesterol (mg/dL)	197.45 ± 37.59	206.49 ± 38.13	0.052
TGs (mg/dL)	102.05 ± 67.34	174.23 ± 67.47	<0.001
HDL-C (mg/dL)	59.00 ± 16.25	48.80 ± 11.81	<0.001
LDL-C (mg/dL)	115.99 ± 32.15	127.85 ± 31.96	0.012
Iron-related biomarkers			
Hb (gm/dL)	14.55 ± 2.41	15.66 ± 3.12	0.003
Iron (µg/dL)	102.97 ± 38.63	107.13 ± 34.14	0.632
Hepcidin (ng/mL)	118.61 ± 93.54	259.77 ± 67.59	<0.001
Ferritin (ng/mL)	117.16 ± 121.84	263.64 ± 169.26	<0.001
TS (%)	29.69 ± 12.62	30.33 ± 10.31	0.765
Elevated hepcidin (*n*, %)	38 (23.6)	46 (97.9)	<0.001
Anemia (*n*, %)	13 (8.1)	2 (4.3)	0.373
Iron-deficiency anemia (*n*, %)	14 (8.7)	0 (0.0)	0.036
Hyperferritinemia (*n*, %)	8 (5.0)	26 (55.3)	<0.001
Liver injury and oxidative stress biomarkers			
Nitrite oxide (μM)	44.44 ± 28.01	61.67 ± 24.33	<0.001
ALT (U/L)	26.14 ± 17.88	54.96 ± 37.98	<0.001
MDA (μM)	40.13 ± 27.54	47.89 ± 21.23	0.005

DIM was defined by the presence of at least four of the following five criteria: elevated serum hepcidin (>200 ng/mL in males and >140 ng/mL in females), hyperferritinemia (serum ferritin > 300 ng/mL in males and >200 ng/mL in females), central obesity (>90 cm in males and >80 cm in females), non-alcoholic fatty liver disease (NAFLD), and at least two abnormal metabolic profiles. Continuous data are presented as the mean ± standard deviation; categorical data are presented as numbers (percentages); * The *p* value was analyzed using the Mann–Whitney test for continuous variables and chi-squared test for categorical variables. MetS, metabolic syndrome; BMI, body-mass index; HbA1C, glycated hemoglobin; TGs, triglycerides; HDL-C, high-density lipoprotein-cholesterol; LDL-C, low-density lipoprotein-cholesterol; Hb, hemoglobin; TS, transferrin saturation; ALT, alanine aminotransferase; MDA, malondialdehyde.

**Table 2:** Multivariate logistic regression of risk factors associated with dysregulated iron metabolism (*N* = 208).

Variable	Univariate					Multivariate *			
	OR	95% CI		*p* Value		OR	95% CI		*p* Value
Age (years)	1.044	1.02	1.07		0.002	1.036	0.977	1.099	0.235
Sex (*n*, %)									
Female	Ref								
Male	1.031	0.54	1.97		0.928				
BMI (kg/m^2^)	1.224	1.13	1.32		<0.001				
Hemoglobin (g/dL)	1.159	1.03	1.31		0.014				
Iron (μg/dL)	1.003	0.99	1.01		0.504				
Hepcidin (ng/mL)	1.02	1.01	1.02		<0.001	1.018	1.010	1.025	<0.001
Ferritin (ng/mL)	1.01	1.00	1.01		<0.001	1.007	1.002	1.012	0.005
Transferrin saturation (%)	1.01	0.98	1.03		0.753				
ALT (U/L)	1.04	1.03	1.06		<0.001	1.029	1.004	1.055	0.021
MDA (μM)	1.01	0.998	1.02		0.115				
Total cholesterol (mg/dL)	1.01	0.998	1.02		0.151				
Triglycerides (mg/dL)	1.01	1.01	1.02		<0.001	1.003	0.993	1.012	0.582
LDL-C (mg/dL)	1.0	1.00	1.02		0.029				
HDL-C (mg/dL)	0.944	0.916	0.97		<0.001	0.929	0.865	0.999	0.046
Fasting blood glucose (mg/dL)	1.02	1.00	1.03		0.023				
Insulin (mIU/mL)	0.983	0.906	1.07		0.679				
HbA1C (%)	1.48	1.07	2.05		0.018	0.689	0.335	1.41	0.309

* The multivariate was adjusted for age, gender, body-mass index (BMI), hepcidin, ferritin, alanine transaminase (ALT), triglycerides, HbA1c, and high-density lipoprotein cholesterol (HDL-C). DIM was defined by the presence of at least four of the following five criteria: elevated serum hepcidin (>200 ng/mL in males and >140 ng/mL in females), elevated serum ferritin (>300 ng/mL in males and >200 ng/mL in females), central obesity (>90 cm in males and >80 cm in females), non-alcoholic fatty liver disease, and at least two abnormal metabolic profiles. Abbreviations: CI, confidence interval; MDA, malondialdehyde; LDL-C, low-density lipoprotein cholesterol.

**Table 3:** Associations of selected response variables with the visceral fat mass and skeletal muscle mass (*N* = 208).

	Visceral Fat Mass (%)					
	^#^ Model 1		* Model 2		^&^ Model 3	
	β (95% CI)	*p* Value	β (95% CI)	*p* Value	β (95% CI)	*p* Value
Hepcidin (ng/mL)	0.003 (0.002–0.005)	<0.001	0.003 (0.002–0.005)	<0.001	0.001 (0.0001–0.002)	0.025
Ferritin (ng/mL)	0.002 (0.0001–0.003)	0.008	0.002 (0.0001–0.003)	0.030	1.187 (−0.001–0.001)	0.972
ALT (U/L)	0.020 (0.014–0.026)	<0.001	0.020 (0.014–0.026)	<0.001	0.004 (0.001–0.007)	0.018
HDL (mg/dL)	−0.030 (−0.041–−0.020)	<0.001	−0.032 (−0.044–−0.021)	<0.001	−0.005 (−0.011–0.001)	0.109
	**Skeletal muscle mass (%)**					
Hepcidin (ng/mL)	−0.003 (−0.011–0.004)	0.388	−0.014 (−0.021–−0.006)	<0.001	−0.004 (−0.008–−0.0001)	0.033
Ferritin (ng/mL)	0.005 (−0.001–0.010)	0.102	−0.006 (−0.012–0.0001)	0.039	−6.395 (−0.003–0.003)	0.968
ALT (U/L)	−0.067 (−0.097–−0.038)	<0.001	−0.084 (−0.110–−0.059)	<0.001	−0.020 (−0.035–−0.004)	0.012
HDL (mg/dL)	0.045 (−0.007–0.097)	0.087	0.142 (0.094–0.191)	<0.001	0.032 (0.004–0.060)	0.027

^#^ Model 1 was adjusted for age; * Model 2 was adjusted for age and gender; ^&^ Model 3 was adjusted for age, gender, and body-mass index. ALT, alanine aminotransferase; HDL, high-density lipoprotein.

**Table 4:** Food groups that were strongly associated with the dysregulated iron metabolism-related dietary pattern scores identified using a reduced rank regression (RRR).

Food Groups	Explained Variation (%)	Factor Loading *
Deep-fried foods	15.87	0.41
Processed meats	11.08	0.34
Chicken and pork	7.88	0.29
Eating out	7.33	0.28
Coffee	4.85	0.23
Animal fat/skin	4.80	0.22
Steamed/boiled/raw food	7.57	−0.28
Dairy products	5.36	−0.24
Total explained variation (%):	64.73	

* Factor loadings show correlations between food groups and the first dietary pattern scores (correlation coefficient for the RRR-derived pattern ≥ |0.20|).

**Table 5:** Clinical characteristics and blood biochemical levels of the study population stratified by tertiles of dietary pattern scores.

Variable	Tertile of Dietary Pattern Scores ^$^			* *p* for Trend	^#^*p* Value
	T1 (*N* = 69)	T2 (*N* = 69)	T3 (*N* = 69)		
Age (years)	41.32 ± 13.80	41.04 ± 13.13	43.81 ± 10.86	0.730	0.752
Sex (*n*, %)					
Male	24 (34.8)	37 (53.6)	44 (63.8)	0.003	0.002
Female	45 (65.2)	32 (46.4)	25 (36.2)		
NAFLD (*n*, %)	56 (81.2)	55 (79.7)	58 (84.1)	0.798	1.000
Metabolic syndrome (*n*, %)	10 (14.5)	18 (26.1)	22 (31.9)	0.052	0.051
Central obesity (*n*, %)	26 (37.7)	32 (46.4)	40 (58.0)	0.057	0.051
Diabetes mellitus (*n*, %)	7 (10.1)	11 (15.9)	7 (10.1)	0.483	1.000
Dyslipidemia (*n*, %)	14 (20.3)	23 (33.3)	23 (33.3)	0.149	0.277
Hypertension (*n*, %)	32 (46.4)	34 (49.3)	37 (53.6)	0.693	1.000
DIM (*n*, %)	9 (13.0)	13 (18.8)	25 (36.2)	0.003	0.003
Abnormal ALT (*n*, %)	7 (10.1)	15 (21.7)	20 (29.0)	0.021	0.018
Anthropometry					
BMI (kg/m^2^)	23.13 ± 5.13	24.83 ± 5.35	25.60 ± 4.62	0.005	0.014
Waist circumference (cm)	81.69 ± 13.13	86.98 ± 13.92	89.41 ± 11.52	0.001	0.002
Male	89.26 ± 12.84	91.72 ± 11.54	91.58 ± 9.80	0.415	1.000
Female	77.65 ± 11.50	81.50 ± 14.59	85.59 ± 13.43	0.016	0.048
Body fat mass (%)	26.86 ± 6.10	27.97 ± 6.12	28.43 ± 6.59	0.142	0.427
Skeletal muscle mass (%)	67.40 ± 6.02	66.30 ± 6.06	65.85 ± 6.53	0.144	0.431
Visceral fat mass (%)	3.27 ± 1.28	3.75 ± 1.33	4.05 ± 1.30	0.001	0.002
Glucose biomarkers					
Fasting blood glucose (mg/dL)	88.88 ± 13.27	92.96 ± 17.93	92.70 ± 23.82	0.236	0.708
Insulin (mIU/mL)	10.02 ± 6.24	8.42 ± 4.41	9.84 ± 5.92	0.894	1.000
HbA1C (%)	5.67 ± 0.69	5.91 ± 1.06	5.88 ± 1.12	0.198	0.595
Lipid biomarkers					
Total cholesterol (mg/dL)	198.84 ± 30.93	199.25 ± 40.46	201.35 ± 41.11	0.697	1.000
Triglyceride (mg/dL)	100.93 ± 71.69	121.74 ± 76.02	132.46 ± 71.40	0.012	0.036
HDL-C (mg/dL)	61.73 ± 16.02	54.10 ± 15.39	54.50 ± 15.34	0.007	0.779
LDL-C (mg/dL)	115.30 ± 28.60	119.81 ± 34.02	121.54 ± 34.24	0.260	0.021
Iron-related biomarkers					
Hemoglobin (g/dL)	14.31 ± 2.57	14.91 ± 2.62	15.24 ± 2.60	0.036	0.109
Iron (μg/dL)	101.75 ± 38.06	107.06 ± 39.20	104.10 ± 34.79	0.713	1.000
Hepcidin (ng/mL)	123.97 ± 93.68	150.94 ± 115.39	178.80 ± 101.92	0.002	0.007
Ferritin (ng/mL)	103.59 ± 130.04	144.32 ± 129.73	204.96 ± 162.65	<0.001	<0.001
Transferrin saturation (%)	28.76 ± 12.17	31.07 ± 12.88	30.03 ± 11.02	0.536	1.000
Elevated serum hepcidin (*n*, %)	12 (17.4)	21 (30.4)	31 (44.9)	0.010	0.007
Anemia (*n*, %)	7 (10.1)	4 (5.8)	3 (4.3)	0.369	0.533
Iron-deficiency anemia (*n*, %)	7 (10.1)	5 (7.2)	1 (1.4)	0.100	0.107
Hyperferritinemia (*n*, %)	5 (7.2)	11 (15.9)	19 (27.5)	0.006	0.004
Liver injury and oxidative stress biomarkers					
Nitrite oxide (μM)	41.97 ± 21.76	54.79 ± 33.36	48.90 ± 26.90	0.193	0.579
ALT (U/L)	25.77 ± 18.71	32.30 ± 24.51	40.14 ± 33.35	0.001	0.004
MDA (μM)	43.41 ± 30.40	38.21 ± 19.32	44.69 ± 28.12	0.798	1.000

Continuous data are presented as the mean ± standard deviation; categorical data are presented as numbers (percentages). * *p* for trend was analyzed by a general linear model for continuous variables and chi-squared test for categorical variables. **^#^** The *p* value was analyzed by a one-way analysis of variance (ANOVA) with the post-hoc Bonferroni correction. ^$^ Tertile of dietary pattern scores: T1 ≤ −0.303; −0.302 ≤ T2 ≤ 0.306; T3 > 0.306. NAFLD, non-alcoholic fatty liver disease; MetS, metabolic syndrome; DIM, dysregulated iron metabolism; BMI, body-mass index; HDL-C, high-density lipoprotein cholesterol; LDL-C, low-density lipoprotein cholesterol; Hb, hemoglobin; TS, transferrin saturation; ALT, alanine aminotransferase; MDA, malondialdehyde.

## Supplementary Materials

The following are available online at https://www.mdpi.com/2072-6643/11/11/2733/s1: Table S1: Strengthening the Reporting of Observational Studies in Epidemiology (STROBE) Statement—Checklist of items that should be included in reports of cross-sectional studies.
