# Supplementary material for: Dysregulated Iron Metabolism-Associated Dietary Pattern Predicts an Altered Body Composition and Metabolic Syndrome
Source: Nutrients. 2019 Nov 11;11(11):2733. doi: 10.3390/nu11112733 (PMC6893840; doi:10.3390/nu11112733)
Supplement: Supplementary file 1 [file nutrients-11-02733-s001.pdf]

Supplementary

Table 1: Strengthening the Reporting of Observational Studies in Epidemiology (STROBE) Statement—Checklist of items that should be included in reports of cross-sectional studies.

	Item No	Recommendation
**Title and abstract**	1	*(a)* Indicate the study's design with a commonly used term in the title or the abstract<br>Page 1: Line 1– 4, Line 25-40
		(b) Provide in the abstract an informative and balanced summary of what was done and what was found<br>What was done: Page 1: Line 26-30. What was found: Page 1, Line 30-38.
Background/rationale	2	Explain the scientific background and rationale for the investigation being reported<br>Page 1: Abstract: Line 25-26; Introduction: page 2: line 82-84
Objectives	3	State specific objectives, including any prespecified hypotheses<br>Page 2: Line 84-88
Study design	4	Present key elements of study design early in the paper<br>Page 2-3: Line 90-106
Setting	5	Describe the setting, locations, and relevant dates, including periods of recruitment, exposure, follow-up, and data collection<br>Page 2: Line 94-97
Participants	6	*(a)* Give the eligibility criteria, and the sources and methods of selection of participants<br>Page 2: Line 94-97
Variables	7	Clearly define all outcomes, exposures, predictors, potential confounders, and effect modifiers. Give diagnostic criteria, if applicable<br>Page 3: Line 119-143
Data sources/ measurement	8*	For each variable of interest, give sources of data and details of methods of assessment (measurement). Describe comparability of assessment methods if there is more than one group<br>Page 3: Line 108-129, Page 4: Line 148-165
Bias	9	Describe any efforts to address potential sources of bias<br>Page 2-3: Line 99-106
Study size	10	Explain how the study size was arrived at<br>Page 2: Line 97-99
Quantitative variables	11	Explain how quantitative variables were handled in the analyses. If applicable, describe which groupings were chosen and why<br>Page 4: Line 166-183
Statistical methods	12	*(a)* Describe all statistical methods, including those used to control for confounding<br>Page 4: Line 166-183

		(b) Describe any methods used to examine subgroups and interactions<br>Page 4: Line 169-172
		(c) Explain how missing data were addressed<br>Page 3: Line 104-105
		(d) If applicable, describe analytical methods taking account of sampling strategy<br>Page 2-3: Line 94-106
		(e) Describe any sensitivity analyses<br>Not applicable
Participants	13*	(a) Report numbers of individuals at each stage of study—eg numbers potentially eligible, examined for eligibility, confirmed eligible, included in the study, completing follow-up, and analysed<br>Page 2-3: Line 94-105
		(b) Give reasons for non-participation at each stage<br>Page 2-3: Line 94-105
		(c) Consider use of a flow diagram<br>Not applicable as patients were mainly recruited in the Division of Gastroenterology and Hepatobiliary Disease, Department of Internal Medicine, Taipei Medical University Hospital (Taipei, Taiwan).<br>Page 2: Line 94-97
Descriptive data	14*	(a) Give characteristics of study participants (eg demographic, clinical, social) and information on exposures and potential confounders<br>Page 5: Line190-201
		(b) Indicate number of participants with missing data for each variable of interest<br>Page 2-3: Line 94-105
Outcome data	15*	Report numbers of outcome events or summary measures<br>Page 6-10: Line 233 – 317.
Main results	16	*(a)* Give unadjusted estimates and, if applicable, confounder-adjusted estimates and their precision (eg, 95% confidence interval). Make clear which confounders were adjusted for and why they were included<br>Page 7 – 8: Line 211-292
		(b) Report category boundaries when continuous variables were categorized<br>Page 8-9: Line 265-275
		(c) If relevant, consider translating estimates of relative risk into absolute risk for a meaningful time period<br>Page 6: lines 217-222, page10: line 280-292
Other analyses	17	Report other analyses done—eg analyses of subgroups and interactions, and sensitivity analyses<br>Not applicable.
Key results	18	Summarise key results with reference to study objectives<br>Page 1: Line 30-38, Page 10: Line 294-304

Limitations	19	Discuss limitations of the study, taking into account sources of potential bias or imprecision. Discuss both direction and magnitude of any potential bias<br>Page 12: Line 377-389
Interpretation	20	Give a cautious overall interpretation of results considering objectives, limitations, multiplicity of analyses, results from similar studies, and other relevant evidence<br>Page 1: Line 315-325, Page 11-12: Line 340-362, Page 13: Line 363-375
Generalisability	21	Discuss the generalisability (external validity) of the study results<br>Page 11: Line 340-362
Funding	22	Give the source of funding and the role of the funders for the present study and, if applicable, for the original study on which the present article is based<br>Page 12: Line 400-402
